# The 3.6-Ma aridity and westerlies history over midlatitude Asia linked with global climatic cooling

**DOI:** 10.1073/pnas.1922710117

**Published:** 2020-09-21

**Authors:** Xiaomin Fang, Zhisheng An, Steven C. Clemens, Jinbo Zan, Zhengguo Shi, Shengli Yang, Wenxia Han

**Affiliations:** ^a^Center for Excellence in Tibetan Plateau Earth Sciences, Chinese Academy of Sciences, 100101 Beijing, China;; ^b^Key Laboratory of Continental Collision and Plateau Uplift, Institute of Tibetan Plateau Research, Chinese Academy of Sciences, 100101 Beijing, China;; ^c^State Key Laboratory of Loess and Quaternary Geology, Institute of Earth Environment, Chinese Academy of Sciences, 710075 Xi’an, China;; ^d^Center for Excellence in Quaternary Science and Global Change, Chinese Academy of Sciences, 710061 Xi’an, China;; ^e^Interdisciplinary Research Center of Earth Science Frontier, Beijing Normal University, 100875 Beijing, China;; ^f^Earth, Environmental, and Planetary Sciences, Brown University, Providence, RI 02912;; ^g^Open Studio for Oceanic-Continental Climate and Environment Changes, Pilot National Laboratory for Marine Science and Technology (Qingdao), 266061 Qingdao, China;; ^h^Key Laboratory of Western China’s Environmental Systems (Ministry of Education), College of Earth and Environmental Sciences, Lanzhou University, 730000 Lanzhou, China;; ^i^Shandong Provincial Key Laboratory of Water and Soil Conservation & Environmental Protection, School of Resource and Environmental Sciences, Linyi University, 276000 Linyi, China

**Keywords:** dust emission, Taklimakan loess sequence, Asian inland aridification, global cooling, Plio-Quaternary

## Abstract

Midlatitude Asia (MLA), strongly influenced by westerlies-controlled climate, is a key source of global atmospheric dust, and plays a significant role in Earth’s climate system . However, it remains unclear how the westerlies, MLA aridity, and dust flux from this region evolved over time. Here, we report a unique high-resolution eolian dust record covering the past 3.6 Ma, retrieved from the thickest loess borehole sequence (671 m) recovered to date, at the southern margin of the Taklimakan desert in the MLA interior. The results show that eolian dust accumulation, which is closely related to aridity and the westerlies, indicates existence of a dry climate, desert area, and stable land surface, promoting continuous loess deposition since at least ∼3.6 Ma. This region experienced long-term stepwise drying at ∼2.7, 1.1, and 0.5 Ma, coeval with a dominant periodicity shift from 41-ka cyclicity to 100-ka cyclicity between 1.1 Ma and 0.5 Ma. These features match well with global ice volume variability both in the time and frequency domains (including the Mid-Pleistocene Transition), highlighting global cooling-forced aridity and westerlies climate changes on these timescales. Numerical modeling demonstrates that global cooling can dry MLA and intensify the westerlies, which facilitates dust emission and transport, providing an interpretive framework. Increased dust may have promoted positive feedbacks (e.g., decreasing atmospheric CO_2_ concentrations and modulating radiation budgets), contributing to further cooling. Unraveling the long-term evolution of MLA aridity and westerlies climate is an indispensable component of the unfolding mystery of global climate change.

## Significance

We recovered the world’s thickest continuous loess record from the southern margin of the Taklimakan desert, a global-scale dust source area. The continuous high-resolution grain size and flux records of dust emission, reflecting histories of aridity and westerlies climate, indicate an extant dry climate, desert area, and stable land surface supporting continuous loess deposition at least since ∼3.6 Ma, and that global cooling, rather than Tibet uplift, modulated the histories of aridity and westerlies climate changes in inland Asia since ∼3.6 Ma. Moreover, our study may suggest potential positive linkages and feedback among dust emission, marine biogeochemical activity, atmospheric CO_2_, and global cooling, which might provide insights into dynamics of Earth’s climate system and improve predictions for the future.

**Fig. 1.**
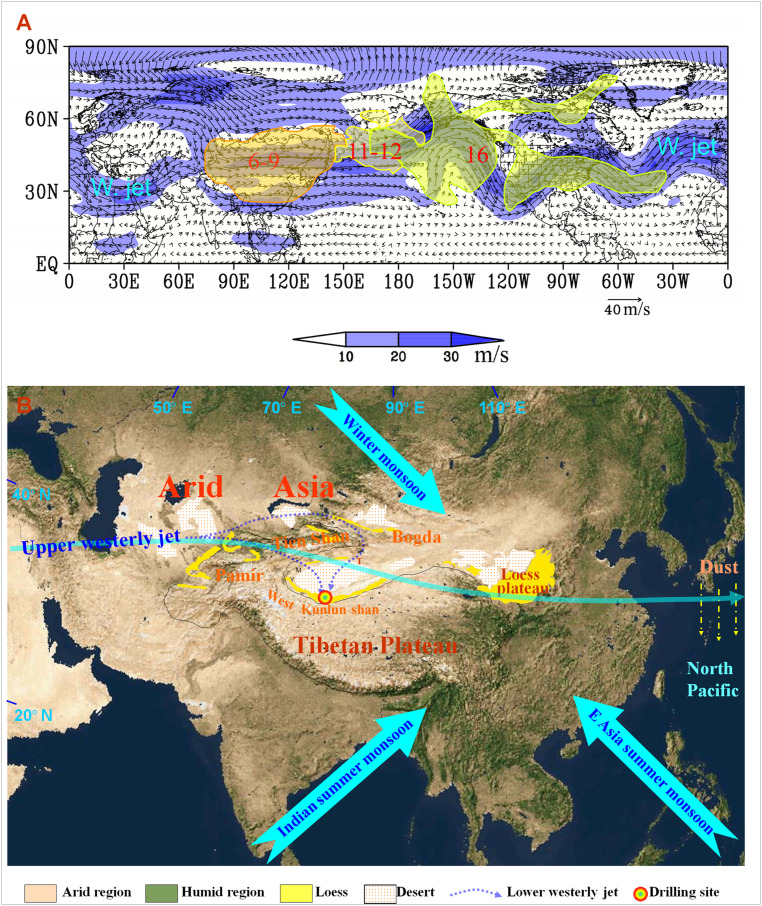
(*A*) The long-distance transport of a dust storm in NH MLA on April 6–9, 2001, via the westerly jet as indicated by the strongest wind speeds in the 500-hPa map of the potential height and vector winds. Red numbers are dates. Black solid lines stand for the potential height, and arrows stand for wind direction (modified from ref. 8). (*B*) Physical geography and schematic atmospheric circulation pattern of Asia with the locations of the loess drilling site. Note that MLA is the largest arid interior region in a temperate continent (pale area) and that East Asia is characterized by a monsoonal humid region (green area). The solid black line outlines the 3,000-m-altitude region.

The vast arid regions in midlatitude Asia (MLA) are among the most prominent landscapes on Earth’s surface, spanning over nine countries and hosting about 0.6 billion people, most suffering from poverty exacerbated by increasing environmental stress due to aridification. Aridity in MLA has long been thought to have strong impacts on Pacific Ocean primary productivity, global geochemical cycling, and climate change by influencing the global radiation budget (1–4) and atmospheric CO_2_ variability (5–7) (Fig. 1*A*). Uplift of the Tibetan Plateau and global cooling have been thought to control the aridification of the MLA interior, and global climates as well, through changes in the westerlies (9–11). However, lack of detailed aridity records hinders our understanding of which mechanisms drive MLA aridification and changes of the westerly jet, and hence the linkages between dust emission from the MLA interior and Pacific Ocean biogeochemical processes and global cooling.

The Tarim basin (560,000 km^2^) in northwest China (Fig. 1*B*), containing the world's largest active dune field, the Taklimakan desert, provides nearly two-thirds of the total dust generated in the MLA interior (1), with approximately half of the <20 μm fine particles being transported by the upper-level westerly jet out of the region to the Pacific Ocean and beyond (1), even joining the global circulation (2, 4, 8) (Fig. 1*A*). Most of the coarse fraction is transported by the lower-level westerly winds generally southward and deposited proximally, chiefly along the central southern Taklimakan desert and on the northern slope of the West Kunlun Shan (Mountains), accumulating as thick loess deposits carpeting various geomorphic surfaces (mountain slopes, foothill fans, river terraces, and basin rims) (12–14) (Fig. 1*B*). Both modern meteorological data and numerical modeling demonstrate clearly that both the lower and upper winds over the Tarim basin are controlled chiefly by the westerlies under modern and peak glacial conditions (Fig. 1*A* and *SI Appendix*, Fig. S1). This loess, like that in the Chinese Loess Plateau, bears a wealth of direct information about the MLA aridification and westerlies change.

The thickest and most continuous loess is found widely distributed across almost all geomorphic surfaces between the altitudes of 2,500 and 4,500 m (12) around the Hetian−Yutian area to the south of the basin, with its core showing the highest stable platforms with loess surface elevations of ∼3,300 m to 3,200 m, ∼2,000 m above the basin floor (*SI Appendix*, Fig. S2). The current circulation, dust storm track/deposition, and dust−loess geochemical and geological comparative analyses show that the lower westerly winds flow over the lower divides of the Pamir and through the Turpan Wind Pass, between the Tian Shan and the Bogda Mountains (15), generating and carrying dust from the desert to the southern rim of the basin and the northern slope of the West Kunlun Shan, forming a continuous loess deposit (12, 16, 17) (Fig. 1*B*). Thus, this loess deposit archives the continuous histories of change in the westerly climate, the drying of the Asian interior, and dust emission in the Tarim basin. Analysis of these sections will shed light on whether these deposits are driven by the uplift of the Tibetan Plateau and contribute to and impact global climatic change.

## Results and Discussion

Few studies have been carried out on the loess deposits from the lower terraces in the southern Tarim basin, due to the scarcity of good outcrops, the remote location, and the sparse attention that has been paid to the dust emission history of the major contributor of the MLA dust budget. In 2006, we carried out a drilling program on the highest fan surface, with the loess platform surface elevation at 3,300 m, at the central southern margin of the Tarim basin (36°11′58.4″N, 81°20′15.4″E). The drilling extended down to 207 m depth. The paleomagnetism of the core determined its basal age to be ∼1 Ma (18). We recently drilled a second core at the same site using a more powerful drilling rig. This latter campaign completely penetrated the loess sequence and reached the fan surface of the Xiyu (Conglomerate) Formation at the depth of 671 m (*SI Appendix*, Fig. S2), with a recovery of 96.1%. This drilling provides evidence that central Asia has a thick loess sequence, which is 2 to 3 times thicker than the loess on the Chinese Loess Plateau and is the thickest known in the world.

**Fig. 2.**
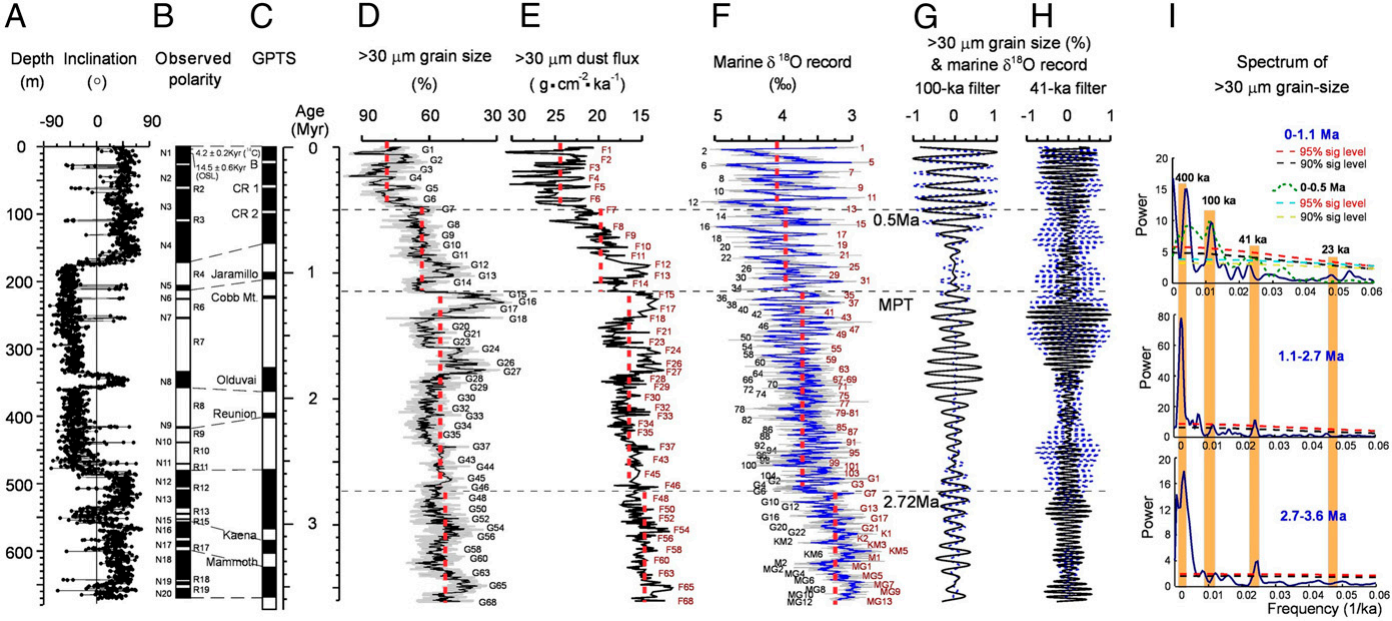
Correlation of the magnetostratigraphy of the loess core in the southern margin of the Tarim basin (*A* and *B*) with the (*C*) GPTS (20, 21), and comparisons of the astronomically tuned time series of the coarse size fraction of >30 μm and its flux in the loess core (*D* and *E*) with the stack of the global marine oxygen isotopes (22) in time domain (*F*) and in frequency domain, filtered for the 100-ka and 41-ka periodicities (*G* and *H*) and >30 μm grain size spectral analysis (*I*). The heavy dashed red lines in *D*–*F* indicate the average values. The dashed blue and solid black lines in *G* and *H* indicate the marine oxygen isotope and >30 μm grain size, respectively. B, Blake event; CR1, Calabrian Ridge 1 event; CR2, Calabrian Ridge 2 event; G1, major grain size peak; F1, major dust flux peak. AF demagnetization and >30 μm coarse size fraction data of uppermost 200 m are taken from the first core (18, 23, 24).

This loess deposit shows similar lithologic characteristics to those we see on the Loess Plateau (*SI Appendix*, Figs. S2–S4). The lithology of the loess core shows a much coarser grain size and much weaker paleosols than those on the central Loess Plateau (19), as the location is much closer to the desert source area, and climate is much drier. The loess is light-yellowish, sandy silt, massive, homogeneous, and loose. In comparison, the paleosols show slightly darker color, less sandy silt (harder), and some biological root channels and pores as well as small white carbonate spots (*SI Appendix*, Fig. S3). High-resolution paleomagnetism determined that the loess sequence was formed from ∼3.6 Ma to the present (*Methods*) (Fig. 2 *A*–*C* and *SI Appendix*, Figs. S5 and S6).

To sustain a large area of continuous deposition of thick loess sediments requires a persistent desert source region providing a stable and considerable dust supply, the presence of a prevailing wind system to transport dust, and a stable land surface for loess accumulation (12, 15). Thus, the appearance of the widely distributed continuous West Kunlun Shan loess at ∼3.6 Ma indicates a rapid desiccation of the Tarim basin at this time and an extant desert area (dunes and vast arid desert fans, exposed river flood valleys with overbank deposits, wind-eroded yardangs, exposed lake floor terrain, etc.) in the Tarim basin, although patches and discontinuous loess-like silt deposits were found earlier in some time intervals, that is, ∼5.3 Ma in the fan sandstone and conglomerate below our loess sequence (16). The increase of Asian dust flux in the Late Pliocene represented by the West Kunlun Shan and Tarim loess record seems to be consistent with the eolian records of the North Pacific Ocean (25).

The coarse size fraction (>30 μm) of the loess deposits has been widely used as a proxy of wind strength, while the dust flux is a direct index of the dust emission or aridity in the source area in the Chinese loess studies (19, 26, 27). These proxies have been proven conceptually and in practice and are also sensitive to the strength of the westerly jet and aridity of the Asian interior (12, 28). Field and satellite observations and measurements of grain sizes of modern dust storms in the Taklimakan desert further demonstrate that the grain sizes of the coarse size fraction increase linearly with westerly wind strength as recorded in deposits in the southern basin (27), while the finer size fractions (<20 μm) are mostly transported out of the region by the upper-level westerly jet to more remote regions, such as the Pacific Ocean and even Greenland (2–4, 8). Higher coarse size fraction >30 μm and its flux indicate stronger westerlies and increased aridity (28), and thus stronger dust emissions. Geologic records and numerical modeling demonstrate that expansion of Northern Hemisphere (NH) ice sheets will intensify the strength of the westerly jet and push it southward (29, 30). These changes in the westerly jet can impact the MLA and Chinese Loess Plateau (26, 31) by enhancing the persistent stationary wave of the westerlies along the northern rim of the Tibetan Plateau and drying the region (9, 10) leading to intensified dust storm activity (26, 31). Thus, the proxy indices of dust grain size and flux can also partially reflect the variations in high-latitude NH ice volume. Accordingly, we analyzed the grain size at 10-cm intervals (∼0.5 ka in resolution) and dust flux at various intervals, mostly 0.3 m to 1 m, following standard methods (Fig. 2 *D* and *E* and see *Methods*).

Based on the magnetostratigraphic age constraints, we carried out spectral analyses of the grain size and dust flux records to determine the dominant periodicities. On this basis, we astronomically tuned the records to orbital eccentricity and obliquity (*Methods*) (Fig. 2 *G*–*I* and *SI Appendix*, Figs. S7 and S8).

The astronomically tuned time series of high-resolution coarse size fraction (>30 μm) and the related flux records of the loess core reveal a long-term increasing trend with obvious stepwise increases at ∼2.7, 1.1, and 0.5 Ma, suggesting enhancements of the westerlies and drying climates at those times. The latest (1 Ma) drying trend was also revealed by the grain size record of the first core (18, 23). Spectral analyses of the records indicate that a dominant 41-ka cycle prevailed in the interval of 3.6 Ma to 1.1 Ma and changed through the interval of 1.1 Ma to 0.5 Ma to a dominant 100-ka cycle for the interval of 0.5 Ma to 0 Ma. Another obvious feature of the spectrum is a large contribution from an ∼400-ka cyclicity (Fig. 2 *G*–*I*).

The coarse size fraction and dust flux records of the loess core are well correlated with benthic marine oxygen isotopes since the Mid-Pliocene (22) (Fig. 2 *D*–*F* and *SI Appendix*, Fig. S9). The aridity and westerly climate changes in the MLA they record generally agree well with the global cooling trend, events in the temporal domain, and periodicity shift in the frequency domain (Fig. 2 *D*–*I*). Major coarser grain size and higher dust flux peaks are mostly correlated with glacial stages in the marine oxygen isotope record, while finer grain size and lower dust flux values are correlated with interglacial stages (Fig. 2 *D*–*F* and *SI Appendix*, Fig. S9). In the context of Plio-Quaternary long-term cooling, there existed the first onset of large-scale glaciations in the high latitudes of the NH at ∼2.7 Ma, the occurrence of the so-called Middle Pleistocene Transition (MPT) at ∼1.1 Ma, decreased temperatures of glacial periods after 0.5 Ma, and a distinct shift of the orbital periodicity influence from obliquity-dominated to eccentricity-dominated cycles at ∼0.5 Ma (22, 32). However, the increased amplitudes of the dust grain size and flux at the MPT and 0.5 Ma are comparatively larger than that of the global average marine oxygen isotope values and their indicated temperature drops (Fig. 2).

Therefore, we believe that the general close matches of the Plio-Quaternary dust emissions driven by the intensification of the westerly and drying climates in the MLA interior with global cooling (Fig. 2) demonstrate that the ice volume and global cooling exert a major role in modulating the aridity and westerly climate in MLA that control the MLA dust emissions. We further hypothesize that the increases of high-latitude ice volume in the NH at the MPT and 0.5 Ma, as indicated by the increased ice volume recorded at Deep Sea Drilling Project 607 in the North Atlantic Ocean (32) and the growth of the extensive Laurentide and Scandinavian ice sheets (33) compared to that of global average marine oxygen isotope record (22) (*SI Appendix*, Fig. S9), are responsible for the increased amplitudes of aridity at the MPT and 0.5 Ma. Such increases in grain size and flux are also seen in the Chinese Loess Plateau (34) and northern Pacific Ocean (25) (*SI Appendix*, Fig. S10). This implies that the expansion of NH ice sheets can greatly enhance the strength of the westerlies climate, the jet, and aridity in MLA. Such signals might be further transferred to the Chinese Loess Plateau and North Pacific Ocean through dust conveyed by upper levels of the westerlies (25, 34–37).

Interestingly, our dust records indicate a strong 400-ka cyclicity that is not present in global marine oxygen isotope records, but is present in globally distributed carbon isotope records (35–38) (*SI Appendix*, Fig. S11). The good match of the dust records with those of the North Pacific Ocean may support the hypothesis that the dust and iron addition to the northern Pacific Ocean promotes marine primary productivity and atmospheric CO_2_ and temperature drops (5–7), forming a positive feedback enhancing further global cooling since the Mid-Pliocene. The marine benthic δ^13^C data partially record the atmospheric CO_2_ concentration and have been suggested as an approximate measure of pCO_2_ (39). Decreased ocean benthic δ^13^C records over oceans, indicating decreased global CO_2_ (34–39), correspond generally with increased long-term dust emission in our record. Furthermore, the prominent 400-ka cycle in our dust records matches well with the benthic δ^13^C records in the South China Sea (35–37) and subtropical North Atlantic Ocean (38) (*SI Appendix*, Fig. S11). Both might indeed hint that the MLA dust emission has considerable impact on marine productivity and global CO_2_ drop.

**Fig. 3.**
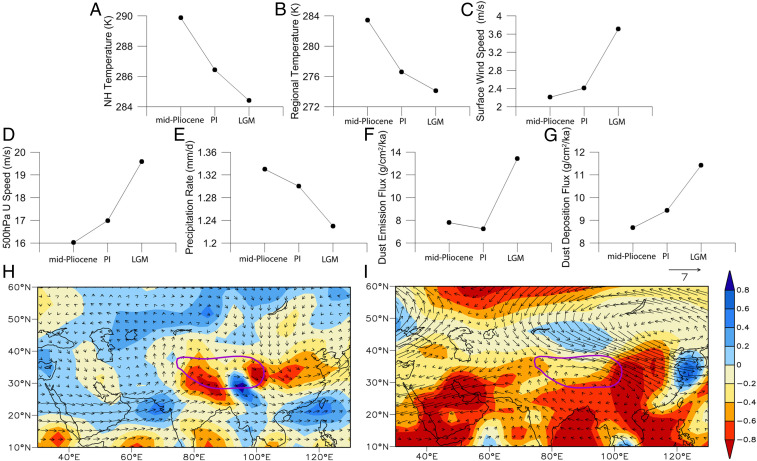
Comparisons of the mean surface temperatures (degrees Celsius; *A*) for the NH, regional temperature (degrees Celsius; *B*), surface wind velocity (meters per second; *C*), >500-hPa westerly wind speed (meters per second; *D*), precipitation rate (millimeters per day; *E*), fine-grained dust emission and deposition fluxes (grams per square centimeter per thousand years; *F* and *G*) averaged for the Taklimakan desert area (75°E to 90°E, 35°N to 45°N) during the Mid-Pliocene, PI, and LGM and the spatial differences in the precipitation rates (millimeters per day) and the 500-hPa westerly wind vectors (meters per second) over Asia between PI and the Mid-Pliocene (*H*) and between the LGM and PI (*I*). The purple lines approximately show the profile of the Tibetan Plateau.

To examine in detail the connections between global cooling factors and the changes of the westerly climate and inland aridity for dust emissions over MLA, we applied the Community Atmosphere Model 3 (CAM3) to conduct a series of numerical simulations for the three typical periods of the Mid-Pliocene (∼3.0 Ma to 3.3 Ma), the preindustrial (PI), and the Last Glacial Maximum (LGM) (*Methods*) (Fig. 3). In the Mid-Pliocene, which had the warmest ocean, the highest CO_2_, and the smallest ice cover of the three periods, the simulated annual temperature over the NH is the highest, with a value of 16.7 °C. This value is warmer than those of the PI and LGM by 3.4 °C and 5.5 °C, respectively (Fig. 3 *A*–*G*). Over the Taklimakan desert region, the variation in the local temperature is similar to the NH mean. In particular, the temperature difference between the Mid-Pliocene and PI is amplified (6.9 °C), indicating that the temperature might be more sensitive to global cooling over the Asian interior. The significant NH cooling from the Mid-Pliocene to the PI and LGM amplified the meridional thermal gradient and thus induced the intensification of the high-level westerly jet. The increased westerly jet, as well as the local surface cooling, both contributed to greater surface wind speeds; especially during the LGM, both the high-level and surface wind speeds are substantially increased due to the high-latitude ice sheets. The annual precipitation rate decreases by 0.10 mm/d from Mid-Pliocene to LGM, which indicates that aridification is enhanced to some extent, although the simulated precipitation is overestimated over this area due to the poor resolution of the Pamir−Tianshan mountains related to relatively coarse model resolution (40). Spatially, compared to the Mid-Pliocene, the climate becomes drier over most regions of northwest China and Mongolia during PI (Fig. 3*H*). The Asian monsoon is also weakened in PI, leading to lower precipitation over nearly the whole of India and eastern China. The high-level westerly wind is slightly strengthened over the Taklimakan desert and becomes significant downwind over northern China and Japan. During the LGM, the precipitation rate is significantly suppressed over the whole of Asia, including both the arid and monsoon regions (Fig. 3*I*). Compared to that of the PI, the westerly wind is obviously intensified by the increased global ice sheets, especially over the Taklimakan and central Asia region. Overall, the modeling results indicate that the enhancements of dust emission and deposition in MLA have close connections with the climatic cooling from the Mid-Pliocene to the LGM (Fig. 3 *F* and *G*), which are mainly facilitated by the strengthened surface wind velocities and high-level westerly wind and inland aridity.

Our loess proxy records and numerical modeling demonstrate that the aridity and westerly climate and thus the dust emissions in MLA have strong connections with global cooling since the Mid-Pliocene. This suggests that global cooling could be the primary forcing needed to dry inland Asia and to enhance the westerlies to form the great Taklimakan desert since the Mid-Pliocene. As well, our results suggest that dust-induced intensified surface albedo, dust reflection, cirrus cloud formation (2), and atmospheric CO_2_ drawdown due to the enhanced primary productivity of microorganisms in the Pacific Ocean [increased availability of dust nutrients (5–7)] might induce positive feedback mechanisms, driving further global cooling.

## Methods

### Paleomagnetic Analysis.

Paleomagnetic block samples were taken at 50-cm intervals in the upper 80 m of the core and at 25-cm intervals in the lower 591 m of the core. In total, 2,500 block samples were obtained. Each of the block samples was cut into three 2 cm × 2 cm × 2 cm cubic subsamples for measurements, forming three sets of specimens. One set of the specimens was subjected to stepwise alternating-field (AF) demagnetization with nine steps of 5, 10, 20, 30, 40, 50, 60, 70, and 80 mT. For cross-checking, the second set of specimens was demagnetized in a Magnetic Measurements Thermal Demagnetizer (MMTD80). Then, all remanent measurements were carried out on a 2G Enterprises Model 760-R cryogenic magnetometer installed in a field-free space (<300 nT) at the Institute of Geology and Geophysics, Chinese Academy of Sciences. No significant difference was found in the characteristic remanent magnetizations (ChRMs) derived from AF demagnetization and thermodemagnetization for most of the samples (*SI Appendix*, Figs. S5 and S6). A total of 2,331 (93%) samples gave reliable ChRMs to determine the magnetostratigraphy.

### Magnetostratigraphic Correlation.

The reliable ChRMs from two sets of samples after removal of the secondary remanent magnetization components through stepwise alternative field demagnetization and thermodemagnetization of secondary remanence are averaged for calculating their inclinations, using a maximum likelihood solution for inclination-only data (41) (*SI Appendix*, Figs. S5 and S6). The depth function of the average inclination determines the 20 normal (N1 to N20) and 19 reversed polarity zones (R1 to R19), which can be readily correlated with the Global Polarity Time Scale (GPTS) (20, 21) (Fig. 2 *A*–*C*). The upper 170 m of the loess core is dominated by normally polarized zones (N1 to N4) and is punctuated by three very short reversals (R1 to R3); the middle part (170 m to 483 m) has mostly reversed polarity zones (R4 to R11) intercalated with some short normal zones (N5 to N11); and the lower part (483 m to 671 m) is again dominated by the normal polarity zones (N12 to N20), with eight short or very short reversals (R12 to R19). The bulk organic ^14^C dating of the top Holocene soil and the optical stimulated luminescence dating of the loess below the Holocene soil yielded ages of ∼4.2 ± 0.2 ka and 14.5 ± 0.6 ka, respectively, demonstrating that the loess sequence is not truncated by erosion (Fig. 2*B*). Thus, we correlate the characteristic polarity zones of the upper, middle, and lower loess cores, with the Brunhes Normal Chron (B), Matuyama Reversed Chron (M), and Gauss Normal Chron (G) and with the B/M boundary at 170 m and the M/G boundary at 483 m (Fig. 2 *A*–*C*). Three very short reversals (R1 to R3) are most likely analogs of the Blake, Calabrian Ridge 1, and Calabrian Ridge 2 events at the ages of 110 ka to 120 ka, 315 ka to 325 ka, and 515 ka to 525 ka (18, 21), respectively. Three short normal zones, N5, N8, and N9, can be well correlated with the Jaramillo, Olduvai, and Reunion events (subchrons), respectively, in the Matuyama Reversed Chron. The short reversed zones R13 to R15 and R17 can be correlated with the Kaena and Mammoth events (subchrons), respectively, in the Gauss Normal Chron. Two short reversed zones—R18 to R19—and many low inclinations values in the bottom of the loess core might be regarded as signals of the approaching boundary of the Gauss Normal Chron and Gilbert Reversed Chron at 3.6 Ma (20) (Fig. 2 *A*–*C*). Extrapolation of the sedimentation rates calculated from the interpreted zones above the Mammoth event yields a similar age for the bottom of the loess core.

### Grain Size and Mass Accumulation Rate.

Grain size analyses were measured at 10-cm intervals using an American Microtrac S3500 laser particle sizer at the Institute of Tibetan Plateau Research, Chinese Academy of Sciences. First, we removed the organic matter from the samples by boiling the sample in a 10% H_2_O_2_ solution for 10 min. We then removed the carbonates from these samples by putting them into a 10% HCl solution for 10 min. Finally, we put the samples into a 3.6% (NaPO_3_)_6_ solution and dispersed them in an ultrasonic bath for 10 min.

Following the formula of An et al. (28), the dust flux of the grain size fraction > 30 μm (grams per square centimeter per thousand years) for the eolian deposits is estimated as

$$\mathrm{dust}\;{\mathrm{flux}}_{>30\mu \text{m}}=f_{\mathrm{eolian}}\times \;\mathrm{SR}\;\times \;\mathrm{DBD,}$$

where *f*_eolian_ is the fraction > 30 μm of the eolian dust in the deposit, SR (centimeters per thousand years) is the dust accumulation rate, and DBD (grams per cubic centimeter) is the dry bulk density.

### Orbital Tuning and Power Spectrum Analysis.

Following the standard orbital tuning method (32, 42, 43), we first obtain an original age sequence for our grain size and dust flux records via a linear interpolation between the paleomagnetic age points (*SI Appendix*, Fig. S7*A*). Spectral analyses of the records based on this time sequence indicate dominant cycles of 41, 100, and 400 ka. The filtered results of the 100-ka and 41-ka bands show distinct similarities with the eccentricity and obliquity cycles in the marine oxygen isotope record (22) and the orbital eccentricity and obliquity cycles (44) (*SI Appendix*, Fig. S7 *B*–*H*). Thus, we established an additional six tie points for the age control based on the visual comparison between our coarse size fraction record and the marine oxygen isotope record (22) aided by rough comparison of their filtered 100-ka and 41-ka cycles with variations of the eccentricity and obliquity cycles (44) (*SI Appendix*, Fig. S7). The final age model of our grain size and dust flux records was obtained by repeatedly tuning our filtered record (centered at 41 ka) based on an initial age model constrained by the paleomagnetic constraints and six tie points to orbital obliquity (44), until the phases and amplitude between our filtered records and those of the orbital records reached maximum fits. We use a phase lag of 8 ka to obliquity (44), following the conventions of previous studies (42, 43, 45) in which the filtered 41-ka component of foraminiferal oxygen isotope data or loess records were considered to lag the NH 65° summer insolation maxima by 7.8 ka at the obliquity band. Orbital tuning was performed using the Match 2.0 program (46). A Gaussian band-pass filter was applied to our record to extract the oscillations with 100-ka and 41-ka periodicities. Data (sampling) resolution is 0.5 ka per point. The detailed method is referred to in our previous publication (43). The final tuning results were presented in *SI Appendix*, Fig. S8. A comparison between the filtered 41-ka components of the coarse size fraction of >30 μm and the flux records of the loess core with the lagged obliquity since 3.6 Ma indicates a good match between the filtered 41-ka component with the lagged phases and amplitude, especially that of the dust flux record (*SI Appendix*, Fig. S8), indicating successful tuning of our grain size and dust flux records to the orbital variation.

### Numerical Experiments.

To examine the effects of the global cooling factors on the inland aridity and westerly jet over Asia, a set of experiments using CAM3 was conducted to represent the global climates of the Mid-Pliocene, the PI, and the LGM. In CAM3, a dust module is enabled (47) to simulate the cycle of eolian dust over Asia. For the Mid-Pliocene (∼3.3 Ma to 3 Ma), the global topography, the ice sheets, and the monthly sea surface temperature (SST) were taken from the Pliocene Research, Interpretation, and Synoptic Mapping 3D dataset (48). The CO_2_ concentration was set to 405 parts per million by volume (ppmv). For the LGM, the ICE-5G data (49) were employed for the cover and topography of the global ice sheets. The monthly SST data were obtained from the Community Climate System Model 3 (CCSM3) LGM experiment by University Cooperation for Atmospheric Research (UCAR), which is available at the Earth System Grid. The CO_2_ concentration was set to 185 ppmv. For the PI, all boundary conditions were kept as those of the present, except the CO_2_ concentration was set to a value of 280 ppmv and the SST was from the CCSM3 PI experiment at UCAR. The orbital parameters for the LGM and PI were calculated by Berger (50). The definition of the dust source areas followed the methods of Shi et al. (51), and the annual precipitation was used to obtain the distributions of the dust sources. These experiments were integrated over 30 y at a horizontal resolution of 2.8° × 2.8°, and the variables for the Asian climate and dust cycle for the last 20 y were averaged to analyze their response to global cooling.

## Supplementary Material

Supplementary File

## Data Availability

There are no data underlying this work.

## References

[1] Zhang X. Y., Arimoto R., An Z. S., Dust emission from Chinese desert sources linked to variations in atmospheric circulation. J. Geophys. Res. 102, 28041–28047 (1997).

[2] Uno I.., Asian dust transported one full circuit around the globe. Nat. Geosci. 2, 557–560 (2009).

[3] Han Y. X., Fang X. M., Xi X. X., Song L. C., Yang S. L., The dust storm in Asia continent and its bio-environmental effects in the North Pacific: A case study of the strongest dust event in April, 2001 in central Asia. Chin. Sci. Bull. 51, 723–730 (2006).

[4] Huang J.., Long-range transport and vertical structure of Asian dust from CALIPSO and surface measurements during PACDEX. J. Geophys. Res. 113, D23212 (2008).

[5] Bishop J. K. B., Davis R. E., Sherman J. T., Robotic observations of dust storm enhancement of carbon biomass in the North Pacific. Science 298, 817–821 (2002). 12399588 10.1126/science.1074961

[6] Watson A. J., Bakker D. C. E., Ridgwell A. J., Boyd P. W., Law C. S., Effect of iron supply on Southern Ocean CO_2_ uptake and implications for glacial atmospheric CO_2_. Nature 407, 730–733 (2000). 11048716 10.1038/35037561

[7] Martin J. H., Glacial-interglacial CO_2_ change: The iron hypothesis. Paleoceanography 5, 1–13 (1990).

[8] Bory A. J.-M., Biscaye P. E., Grousset F. E., Two distinct seasonal Asian source regions for mineral dust deposited in Greenland (NorthGRIP). Geophys. Res. Lett. 30, 1167 (2003).

[9] Kutzbach J. E., Guetter P. J., Ruddiman W. F., Prell W. L., Sensitivity of climate to late Cenozoic uplift in southern Asia and the American west: Numerical experiments. J. Geophys. Res. 94, 18393–18407 (1989).

[10] Manabe S., Broccoli A. J., Mountains and arid climates of middle latitudes. Science 247, 192–195 (1990). 17813285 10.1126/science.247.4939.192

[11] Hao Q.., Delayed build-up of Arctic ice sheets during 400,000-year minima in insolation variability. Nature 490, 393–396 (2012). 23034648 10.1038/nature11493

[12] Fang X. M.., Loess in Kunlun Mountains and its implications on desert development and Tibetan Plateau uplift in west China. Sci. China. Ser. D Earth Sci. 45, 289–299 (2002).

[13] Yang X. P., The grain size of the windborne sediments in the areas of Keriya river (northwestern China) and its paleoenvironmental interpretation. Quat. Sci. 19, 373–379 (1999).

[14] Li B. S.., Geological age of the sand and dust deposits of the Aqiang section in the extremely arid region of China. Acta Geol. Sin. 72, 83–92 (1998).

[15] Bai Z. Y., Xu G. C., Meteorology of Northwest China, (Meteorology, 1988).

[16] Sun J., Liu T., The age of the Taklimakan Desert. Science 312, 1621 (2006). 16778048 10.1126/science.1124616

[17] Zan J. B.., Magnetic properties of surface soils across the southern Tarim Basin and their relationship with climate and source materials. Chin. Sci. Bull. 56, 290–296 (2011).

[18] Zan J. B., Fang X. M., Yang S. L., Nie J. S., Li X., A rock magnetic study of loess from the West Kunlun Mountains. J. Geophys. Res. 115, B10101 (2010).

[19] Ding Z. L.., Coeval changes in grain size and sedimentation rate of eolian loess, the Chinese Loess Plateau. Geophys. Res. Lett. 28, 2097–2100 (2001).

[20] Ogg J. G., Smith A. G., “The geomagnetic polarity time scale” in A Geologic Time Scale 2004, Gradstein F. M., Ogg J. G., Smith A. G., Eds. (Cambridge University Press, 2004), pp. 63–86.

[21] Langereis C. G., Dekkers M. J., de Lange G. J., Paterne M., van Santvoort P. J. M., Magnetostratigraphy and astronomical calibration of the last 1.1 Myr from an eastern Mediterranean piston core and dating of short events in the Brunhes. Geophys. J. Int. 129, 75–94 (1997).

[22] Zachos J., Pagani M., Sloan L., Thomas E., Billups K., Trends, rhythms, and aberrations in global climate 65 Ma to present. Science 292, 686–693 (2001). 11326091 10.1126/science.1059412

[23] Zan J. B., Fang X. M., Yang S. L., Yan M. D., Evolution of the arid climate in High Asia since ∼1 Ma: Evidence from loess deposits on the surface and rims of the Tibetan Plateau. Quat. Int. 313–314, 210–217 (2013).

[24] Zan J. B., “Loess on west Kunlun Mountains and aridification of Asian inland” PhD thesis, Lanzhou University, Lanzhou, China (2010), pp. 47–56.

[25] Rea D. K., Snoeckx H., Joseph L. H., Late Cenozoic eolian deposition in the North Pacific: Asian drying, Tibetan uplift, and cooling of the Northern Hemisphere. Paleoceanography 13, 215–224 (1998).

[26] Porter S. C., An Z. S., Correlation between climate events in the North Atlantic and China during the last glaciation. Nature 375, 305–308 (1995).

[27] Lin Y., Mu G., Xu L., Zhao X., The origin of bimodal grain-size distribution for aeolian deposits. Aeolian Res. 20, 80–88 (2016).

[28] An Z.., Interplay between the Westerlies and Asian monsoon recorded in Lake Qinghai sediments since 32 ka. Sci. Rep. 2, 619 (2012). 22943005 10.1038/srep00619 PMC3431539

[29] Wang N., Jiang D., Lang X., Northern Westerlies during the Last Glacial Maximum: Results from CMIP5 simulations. J. Clim. 31, 1135–1153 (2018).

[30] Laȋné A.., Northern hemisphere storm tracks during the last glacial maximum in the PMIP2 ocean-atmosphere coupled models: Energetic study, seasonal cycle, precipitation. Clim. Dyn. 32, 593–614 (2009).

[31] Shang K., Liu X., Relationship between the sharp decrease in dust storm frequency over East Asia and the abrupt loss of Arctic sea ice in the early 1980s. Geol. Mag. 157, 729–740 (2020).

[32] Raymo M. E., Ruddiman W. F., “DSDP site 607 isotope data and age models” (IGBP PAGES/World Data Center for Paleoclimatology Data Contribution Series #2004-010, National Oceanic and Atmospheric Administration, 2004).

[33] de Boer B., Lourens L. J., van de Wal R. S., Persistent 400,000-year variability of Antarctic ice volume and the carbon cycle is revealed throughout the Plio-Pleistocene. Nat. Commun. 5, 2999 (2014). 24385005 10.1038/ncomms3999

[34] Han Y.., Asian inland wildfires driven by glacial-interglacial climate change. Proc. Natl. Acad. Sci. U.S.A. 117, 5184–5189 (2020). 32094170 10.1073/pnas.1822035117 PMC7071868

[35] Wang P., Tian J., Lourens L. J., Obscuring of long eccentricity cyclicity in Pleistocene oceanic carbon isotope records. Earth Planet. Sci. Lett. 290, 319–330 (2010).

[36] Clemens S. C., Murray D. W., Prell W. L., Nonstationary phase of the Plio-Pleistocene Asian monsoon. Science 274, 943–948 (1996). 8875928 10.1126/science.274.5289.943

[37] Clemens S. C., Prell W. L., Sun Y., Liu Z., Chen G., Southern Hemisphere forcing of Pliocene δ^18^O and the evolution of Indo-Asian monsoons. Paleoceanography 23, PA4210 (2008).

[38] Hodell D. A., Channell J. E. T., Mode transitions in Northern Hemisphere glaciation: Co-evolution of millennial and orbital variability in Quaternary climate. Clim. Past 12, 1805–1828 (2016).

[39] Lisiecki L. E., A benthic δ^13^C–based proxy for atmospheric pCO_2_ over the last 1.5 Myr. Geophys. Res. Lett. 37, L21708 (2010).

[40] Sha Y.., Role of the Tian Shan Mountains and Pamir Plateau in increasing spatiotemporal differentiation of precipitation over interior Asia. J. Clim. 31, 8141–8162 (2018).

[41] Arason Þ., Levi S., Maximum likelihood solution for inclination-only data in paleomagnetism. Geophys. J. Int. 182, 753–771 (2010).

[42] Shackleton N. J., Berger A., Peltier W. R., An alternative astronomical calibration of the lower Pleistocene timescale based on ODP site 677. Trans. R. Soc. Edinb. Earth Sci. 81, 251–261 (1990).

[43] Han W. X., Fang X. M., Berger A., Yin Q. Z., An astronomically tuned 8.1 Ma eolian record from the Chinese Loess Plateau and its implication on the evolution of Asian monsoon. J. Geophys. Res. 116, D24114 (2011).

[44] Laskar J.., A long-term numerical solution for the insolation quantities of the Earth. Astron. Astrophys. 428, 261–285 (2004).

[45] Imbrie J.., “The orbital theory of Pleistocene climate: Support from a revised chronology of the marine δ ^18^O record” in Milankovitch and Climate Part 1, Berger A. L., Ed. . (D. Reidel, 1984), pp. 269–305.

[46] Lisiecki L. E., Lisiecki P. A., Application of dynamic programming to the correlation of paleoclimate records. Paleoceanography 17, 1049 (2002).

[47] Mahowald N.., Change in atmospheric mineral aerosols in response to climate: Last glacial period, preindustrial, modern, and doubled carbon dioxide climates. J. Geophys. Res. 111, D10202 (2006).

[48] Dowsett H. J., Robinson M. M., Foley K. M., Pliocene three dimensional global ocean temperature reconstruction. Clim. Past 5, 769–783 (2009).

[49] Peltier W. R., Global glacial isostasy and the surface of the ice-age Earth: The ICE-5G (VM2) model and GRACE. Annu. Rev. Earth Planet. Sci. 32, 111–149 (2004).

[50] Berger A., Long-term variations of daily insolation and Quaternary climatic changes. J. Atmos. Sci. 35, 2362–2367 (1978).

[51] Shi Z.., Simulated variations of eolian dust from inner Asian deserts at the mid-Pliocene, last glacial maximum, and present day: Contributions from the regional tectonic uplift and global climate change. Clim. Dyn. 37, 2289–2301 (2011).

