# Supplementary material for: The 3.6-Ma aridity and westerlies history over midlatitude Asia linked with global climatic cooling
Source: Proc Natl Acad Sci U S A. 2020 Sep 21;117(40):24729–34. doi: 10.1073/pnas.1922710117 (PMC7547271; doi:10.1073/pnas.1922710117)
Supplement: Supplementary File [file pnas.1922710117.sapp.pdf]

PNAS
www.pnas.org

Supplementary Information for

The 3.6-Ma aridity and westerlies history over midlatitude Asia linked with global climatic cooling

Xiaomin Fang[a,b,c,1], Zhisheng An[c,d,e,1], Steven C. Clemens[f], Jinbo Zan[a,b], Zhengguo Shi[c,g], Shengli Yang[h], and Wenxia Han[i]

[a]Center for Excellence in Tibetan Plateau Earth Sciences, Chinese Academy of Sciences, 100101 Beijing, China;
[b]Key Laboratory of Continental Collision and Plateau Uplift, Institute of Tibetan Plateau Research, Chinese Academy of Sciences, 100101 Beijing, China;
[c]State Key Laboratory of Loess and Quaternary Geology, Institute of Earth Environment, Chinese Academy of Sciences, 710075 Xi'an, China;
[d]Center for Excellence in Quaternary Science and Global Change, Chinese Academy of Sciences, 710061 Xi'an, China;
[e]Interdisciplinary Research Center of Earth Science Frontier, Beijing Normal University, 100875 Beijing, China;
[f]Earth, Environmental, and Planetary Sciences, Brown University, Providence, RI 02912;
[g]Open Studio for Oceanic-Continental Climate and Environment Changes, Pilot National Laboratory for Marine Science and Technology (Qingdao), 266061 Qingdao, China;
[h]Key Laboratory of Western China's Environmental Systems (Ministry of Education), College of Earth and Environmental Sciences, Lanzhou University, 730000 Lanzhou, China;
[i]Shandong Provincial Key Laboratory of Water and Soil Conservation & Environmental Protection, School of Resource and Environmental Sciences, Linyi University, 276000 Linyi, China

[1]To whom correspondence may be addressed.
Email: fangxm@itpcas.ac.cn or anzs@loess.llqg.ac.cn.

This PDF file includes:

Figures S1 to S11
SI References

www.pnas.org/cgi/doi/10.1073/pnas.1922710117

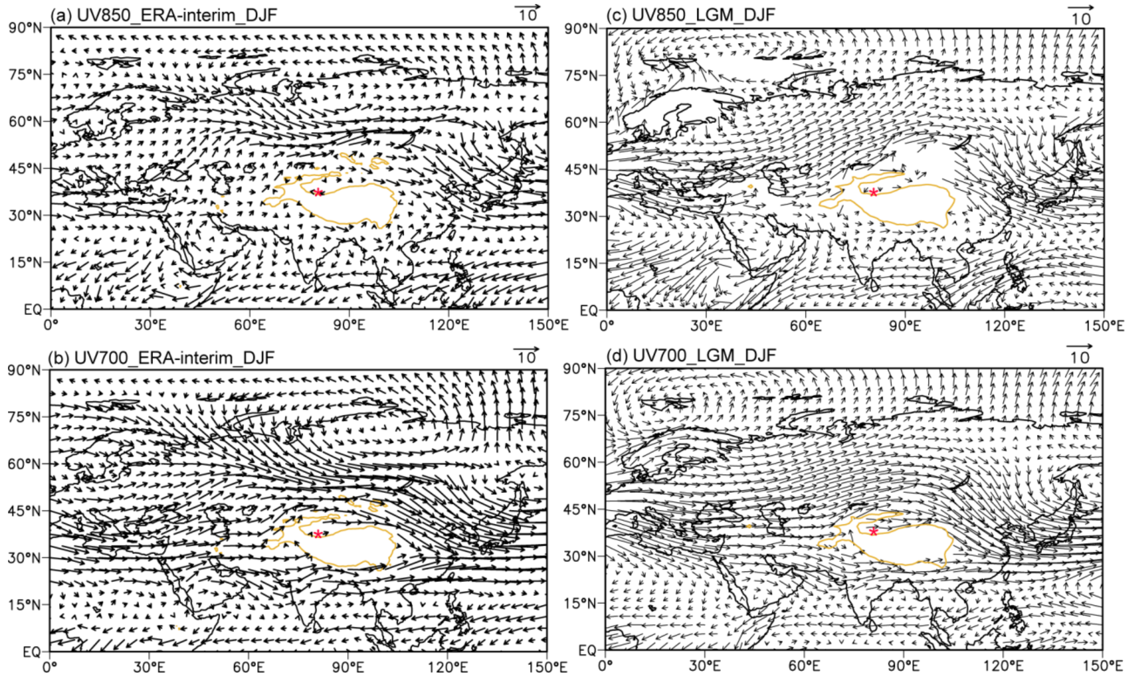

Fig. S1. Modern and Last Glacial Maximum (LGM) 850hPa and 700hPa wind vectors (m/s) in boreal winter based on ERA-interim analysis and CESM experiments. Yellow line shows the orography higher than 2500m. Red star indicates the drilling site of the West Kunlun Shan loess. The LGM data simulated by CESM is from (1).

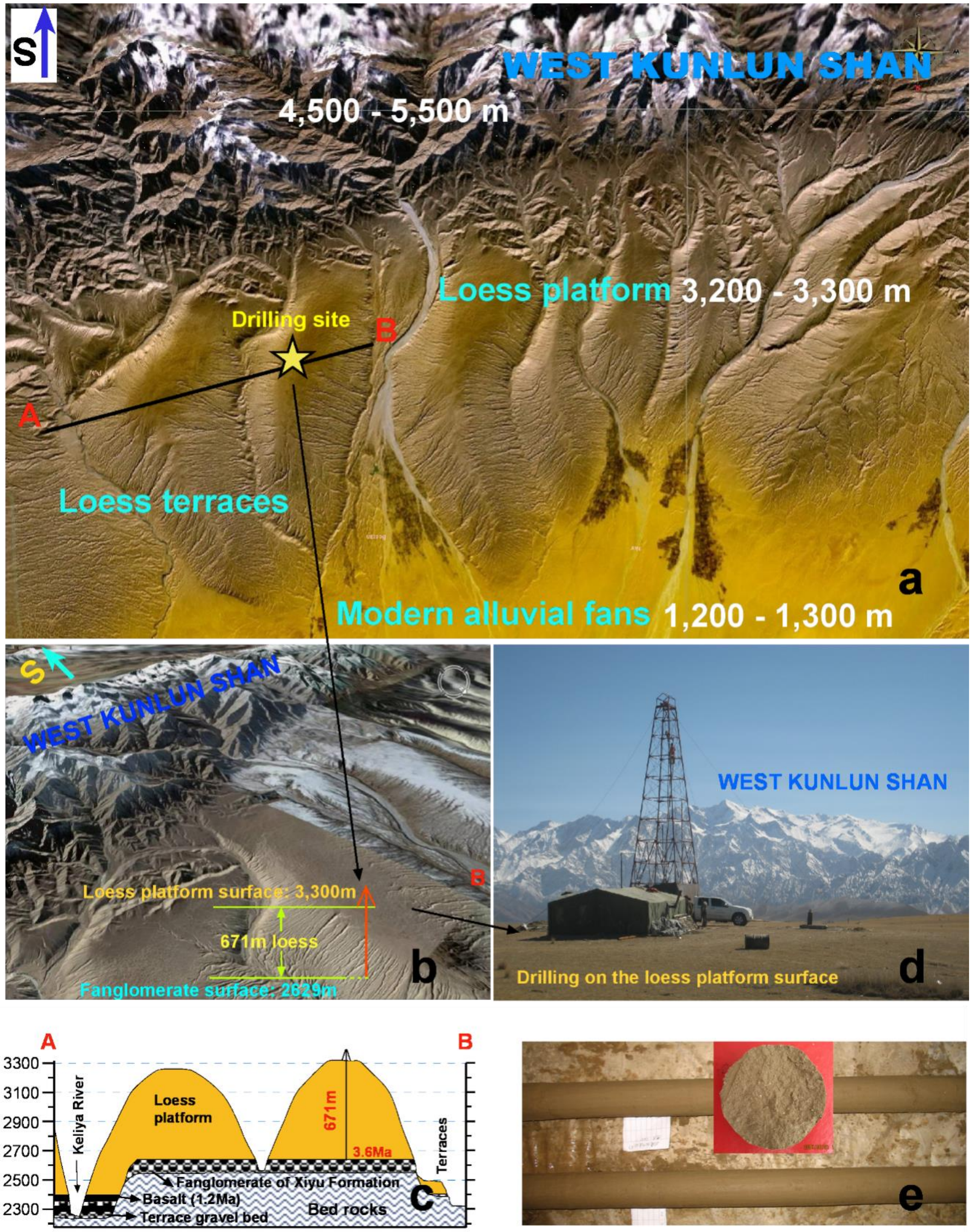

Fig. S2. Geomorphology and loess distribution at the drilling site with the configuration of the drilling setups (See Fig. 1 for location). The schematic geological cross section east-west across the drilling site is shown in the lower left corner, showing that after the deposition of the alluvial conglomerates of the Xiyu Formation, West Kunlun Shan, began a rapid uplift followed by river incision and then by loess deposition at ~3.6 Ma; the subsequent uplift and river incision formed a series of river terraces on which the loess continued to accumulate. Note that one of the terrace gravel beds in the valley of the Keliya River contains two layers of basalt flowing down into the

ancient valley from the West Kunlun Shan with an Ar-Ar dated age of 1.2 Ma. The loess sequence overlying the basalt flow was paleomagnetically dated to approximately 0.8 Ma (2). A view of the drilling field and the obtained high quality of the loess cores are shown in the lower right corner (inset indicates the cross-section of a typical core with a diameter of 8 cm). These photographs demonstrate that the loess deposit shows very light colors, including light yellowish to light brownish yellows, and is characterized by a uniformly loose texture and massive structure, similar to those we see on the Loess Plateau, but it is difficult to clearly identify the paleosol layers in the sequence.

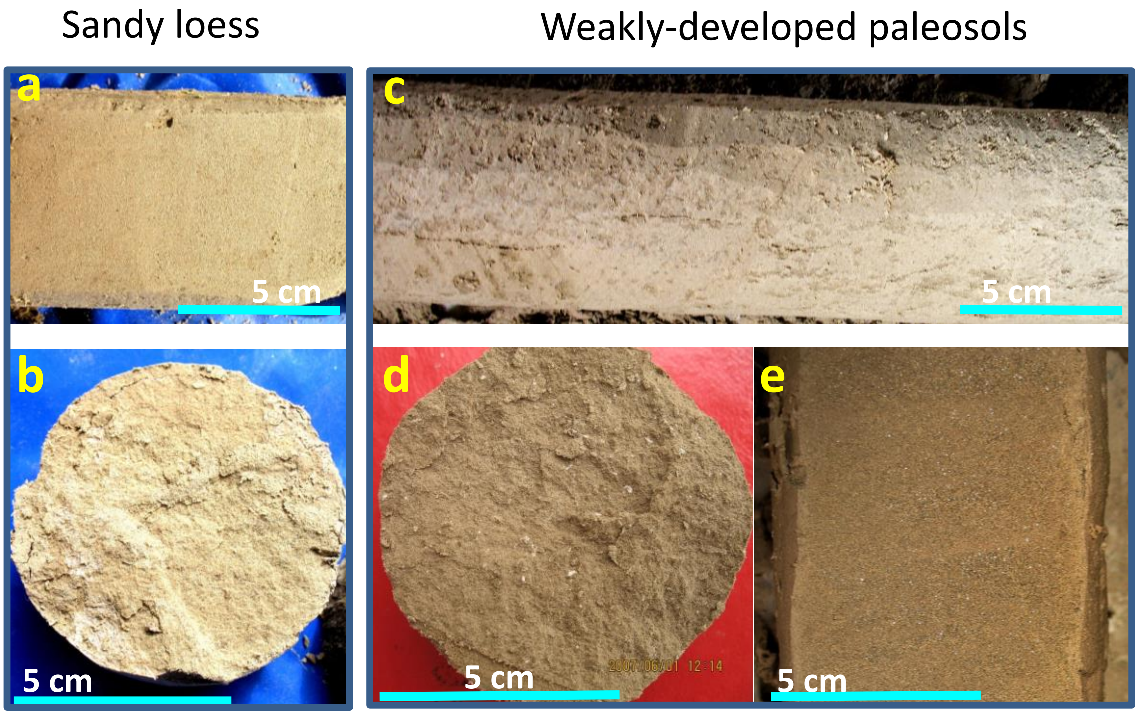

Fig. S3. Field photos showing the lithology and pedofeatures of loess and paleosols in the core of the West Kunlun Shan and southern Tarim loess. Light yellowish, massive, homogeneous and loose sandy loess (a, b). Light brownish yellowish-dull yellowish, massive to weak-blocky paleosols with pedofeatures of some biological channels and pores (c) and white carbonate spots (d, e).

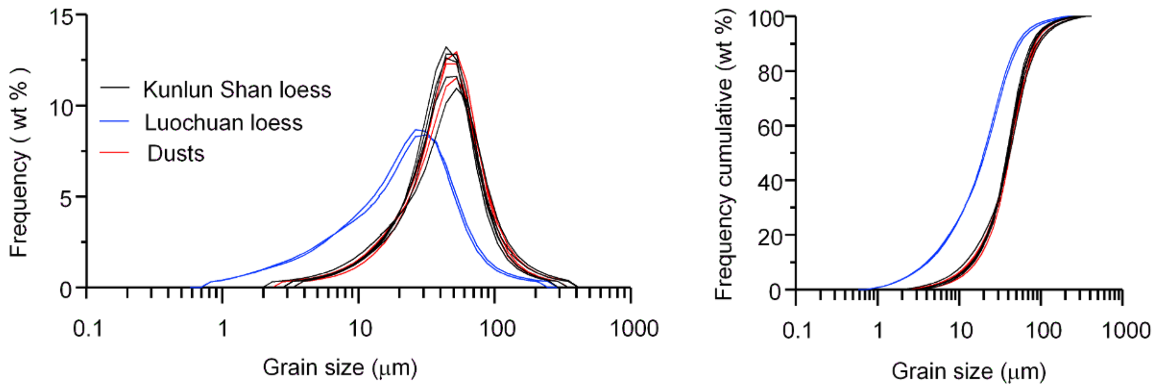

Fig. S4. Grain size distribution of representative samples from the loess core and their comparisons with those of in situ eolian dusts carried by dust storms in the Taklimakan Desert and those of loess from the Chinese Loess Plateau. It is plausible that the convergence of the two branches of lower-level westerly winds over the Pamir pass in the west and the eastern Tian Shan pass in the northeast sweeps the central desert and adds energy to dust transport (2) (Fig. 1). During the drilling operation, frequent dust storms occurred. The grain sizes of some of the dusts collected at the drilling site (~2100 m above the desert surface) show the same grain size composition as that of the loess from the core.

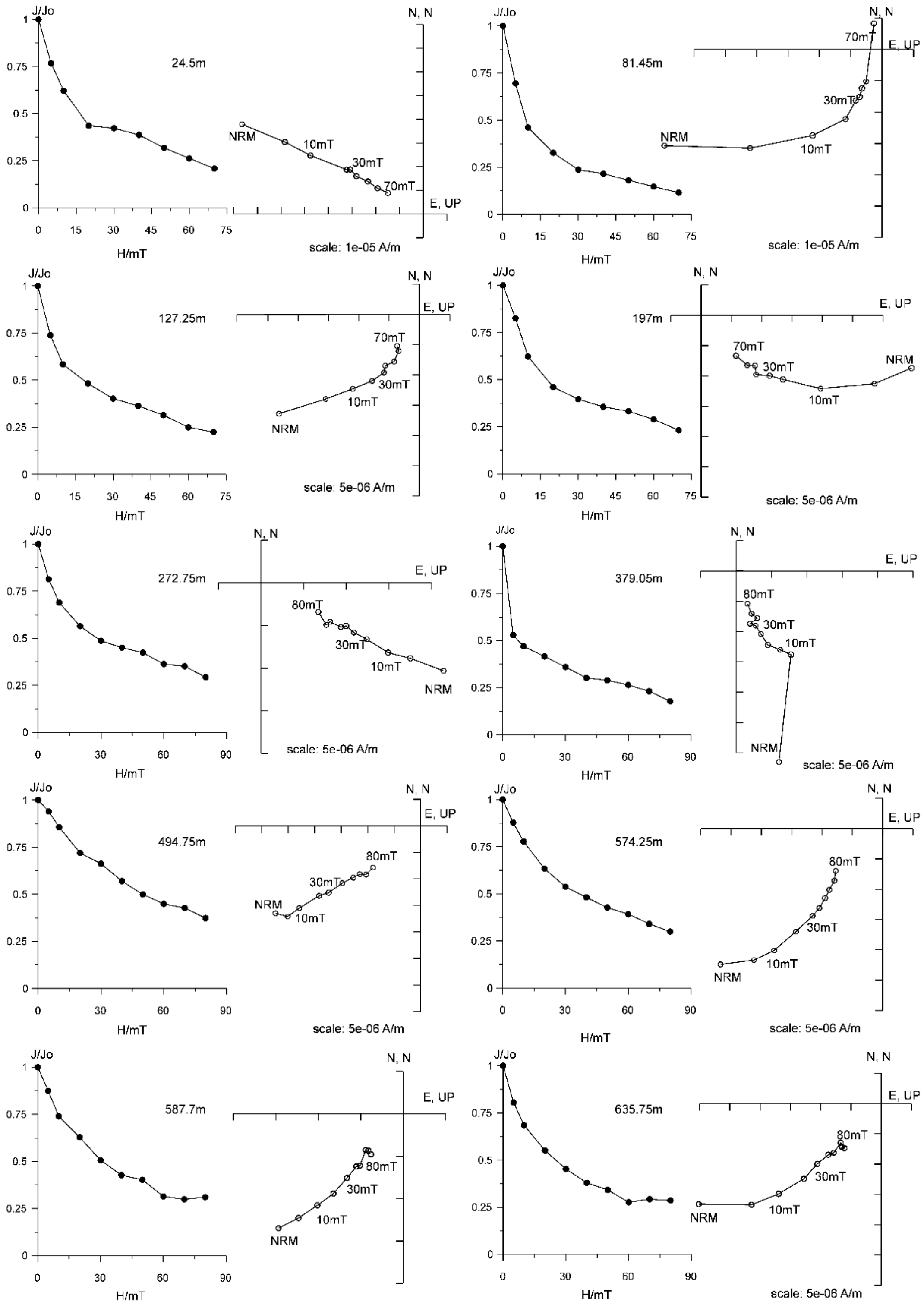

Fig. S5. Orthogonal projections of representative samples via alternating-field (AF) demagnetization. Characteristic remanent magnetizations (ChRMs) are clearly isolated after 30 mT AF-demagnetization. Relatively higher values of ChRMs after 80 mT AF-demagnetization in the samples indicate that some ChRMs are carried by hard magnetic minerals. The open symbols represent vertical projections.

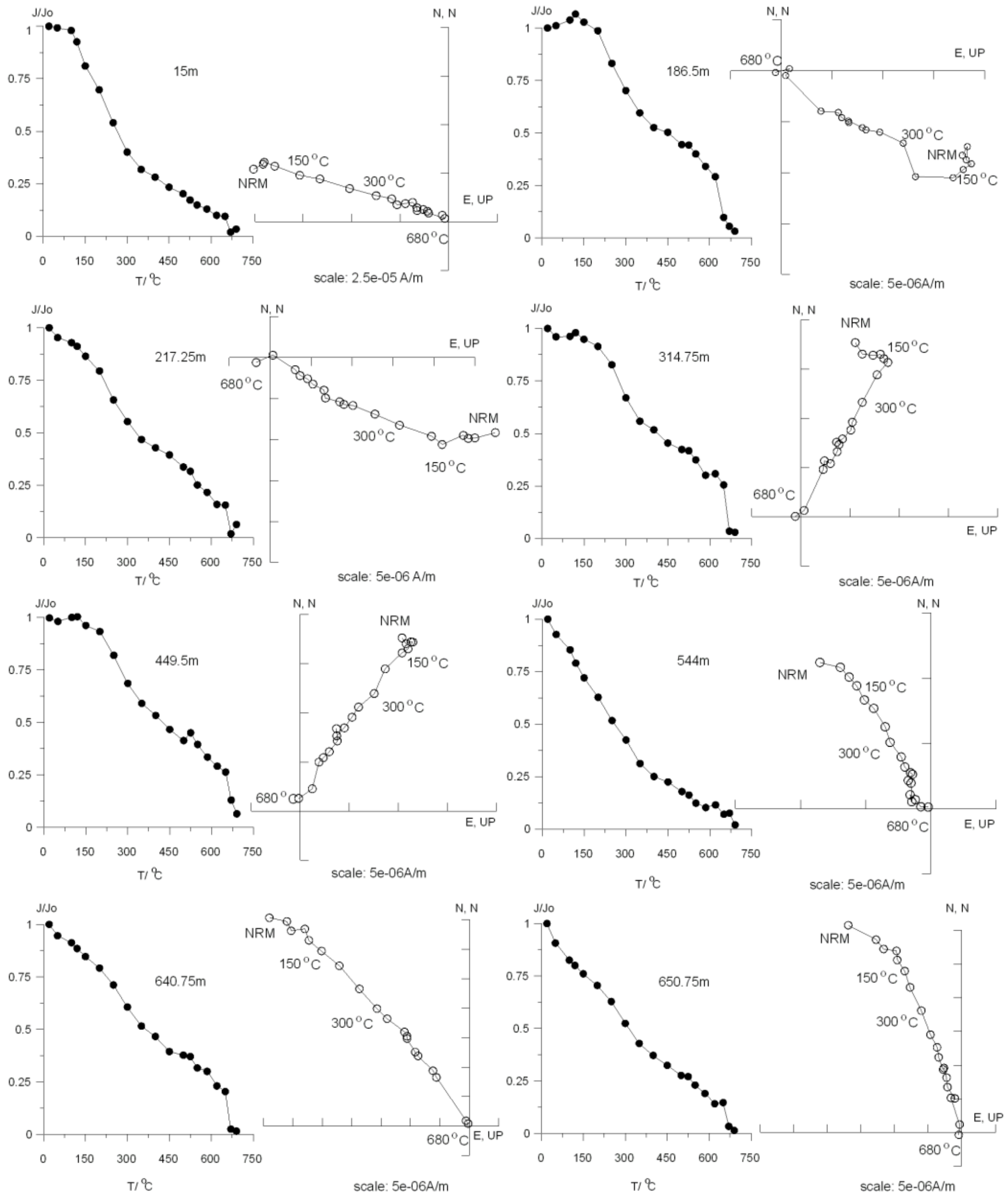

Fig. S6. Orthogonal projections of representative samples via thermal demagnetization. ChRMs are clearly isolated after the 200°C thermal demagnetization. Two clear fast drops between 200°C and 350°C and between 650°C and 680°C demonstrate that maghemite and haematite are most likely the carriers of the ChRMs. The open symbols represent vertical projections.

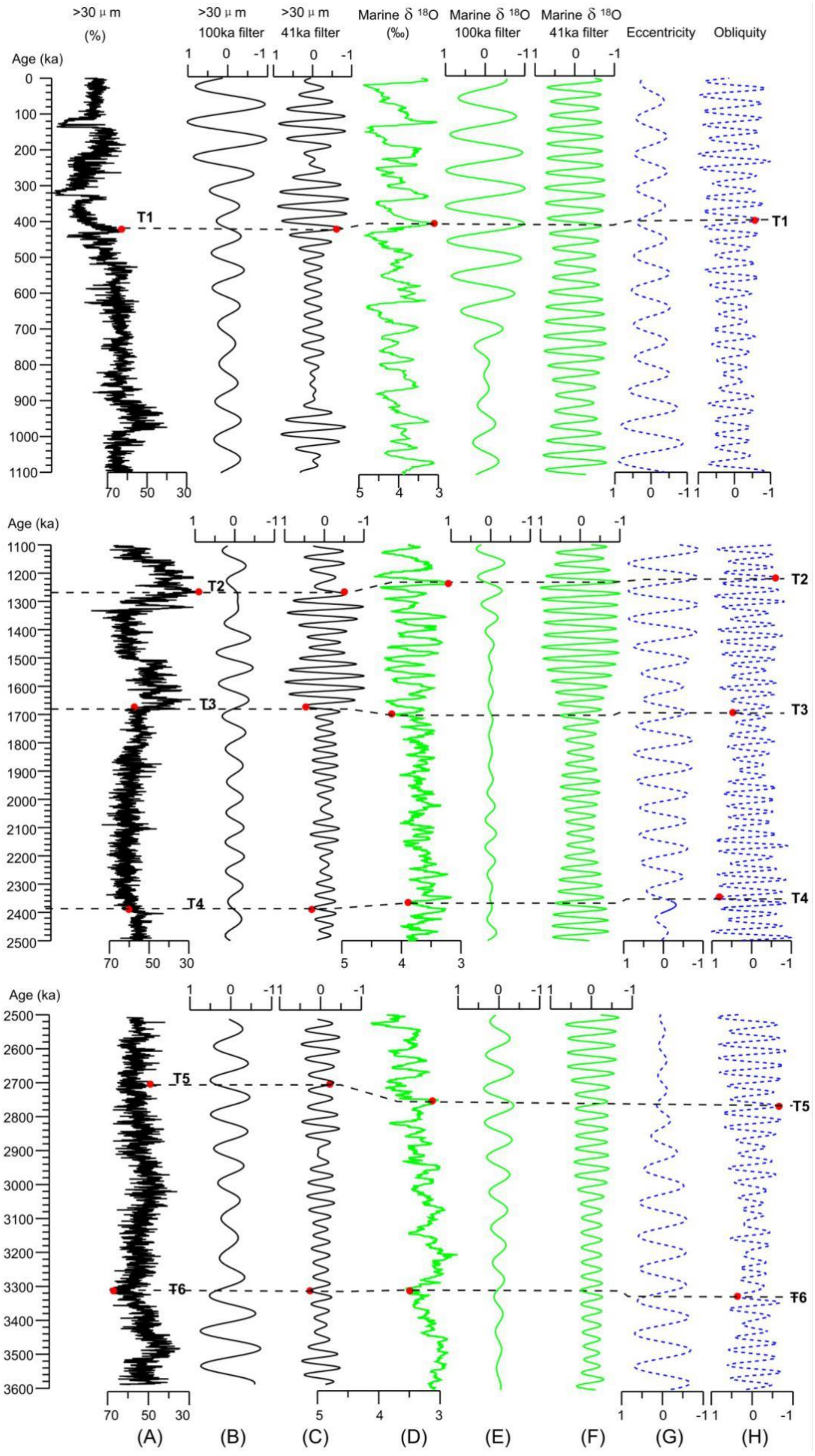

>30 μ m (%)
>30 μ m 100ka filter
>30 μ m 41ka filter
Marine δ 18O (‰)
Marine δ 18O 100ka filter
Marine δ 18O 41ka filter
Eccentricity
Obliquity
Age (ka)
T1
T2
T3
T4
T5
T6
(A)
(B)
(C)
(D)
(E)
(F)
(G)
(H)

Fig. S7. Time series of the coarse size fraction > 30 μm of the grain size (GS) record from the loess core based on paleomagnetic age controls (A) and its 100-ka and 41-ka filtered components (B, C) over the intervals 0–1.1 Ma, 1.1–2.5 Ma and 2.5–3.6 Ma. For comparison, the global marine oxygen isotope record (3) (D) and its filtered components in the 100-ka and 41-ka bands (E, F) and the variations of the eccentricity and obliquity cycles (4) for these intervals are also plotted (G, H). The visual comparison between the coarse size fraction (A) and the global marine oxygen isotope record (3) (D) aided by rough comparison of their filtered 100-ka and 41-ka cycles with variations of the eccentricity and obliquity cycles (4) yielded six preliminary tie points (red spots, T1-T6) for our grain size records. Based on paleomagnetic age controls and these six tie points, we obtained an initial age model. The final age model of our grain size and dust flux records was obtained by repeatedly tuning the filtered 41-ka record based on the initial age model to orbital obliquity until the phases and amplitude between our filtered records and those of the orbital records reached maximum fits.

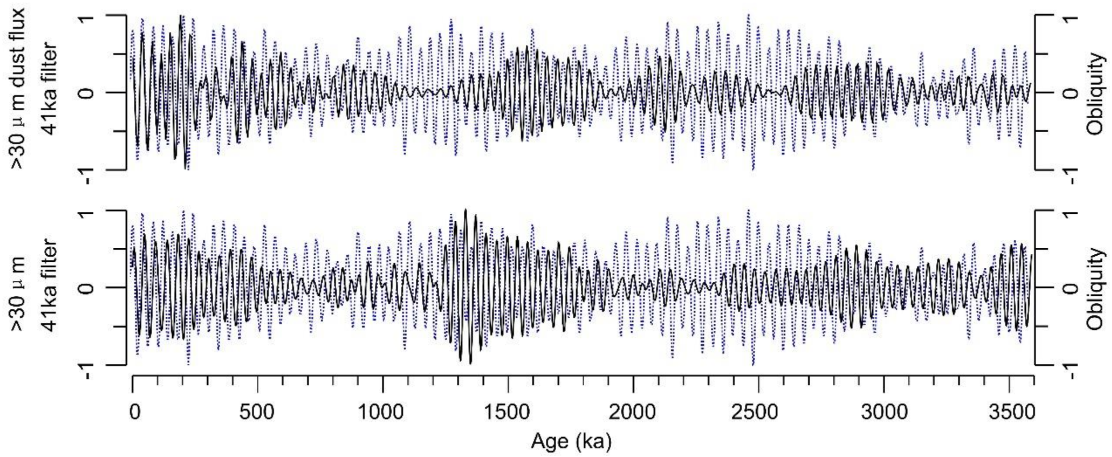

Fig. S8. Comparison of the 41-ka component variation filtered from the grain size records of the loess core (solid line) with the orbital obliquity (4) (8 ka lagged) (blue dashed lines) over the past 3.6 Ma. A Gaussian band filter centered at 0.02439 ka^{-1} was used to identify the 41-ka component.

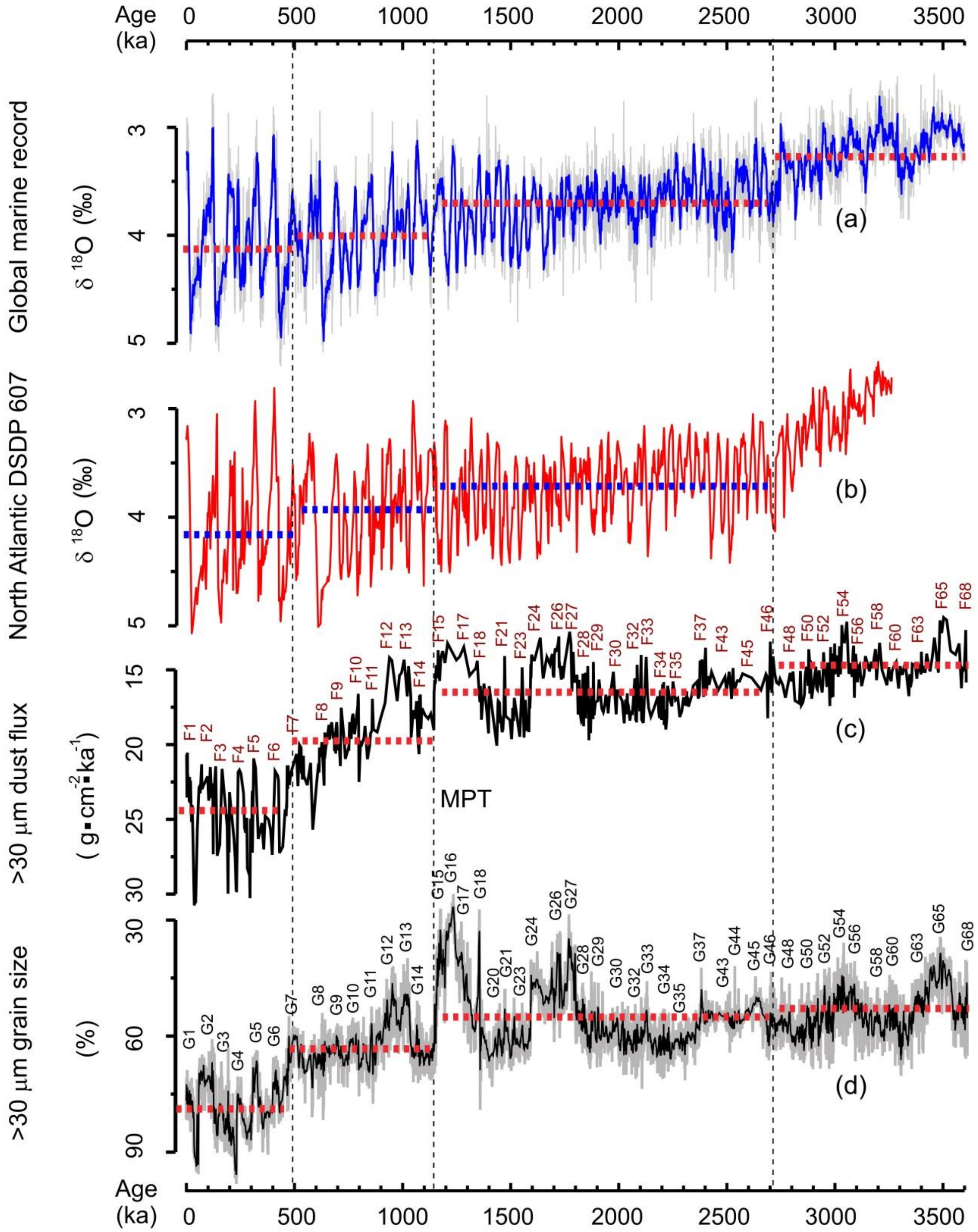

Fig. S9. Comparison of the dust grain size and flux records from the southern Tarim Basin in MLA (c, d) with the oxygen isotope records of the average global marine sediments (3) (a) and core DSDP 607 from the Northern Atlantic Ocean (5) (b). Note the enhanced ice volume of core DSDP 607 compared to the global average at the MPT and 0.5 Ma; this may indicate larger ice caps occurring in the Arctic region at those times, which may be responsible for the enlarged amplitudes of our dust records at those times. G1: Major grain size peak; F1: Major dust flux peak.

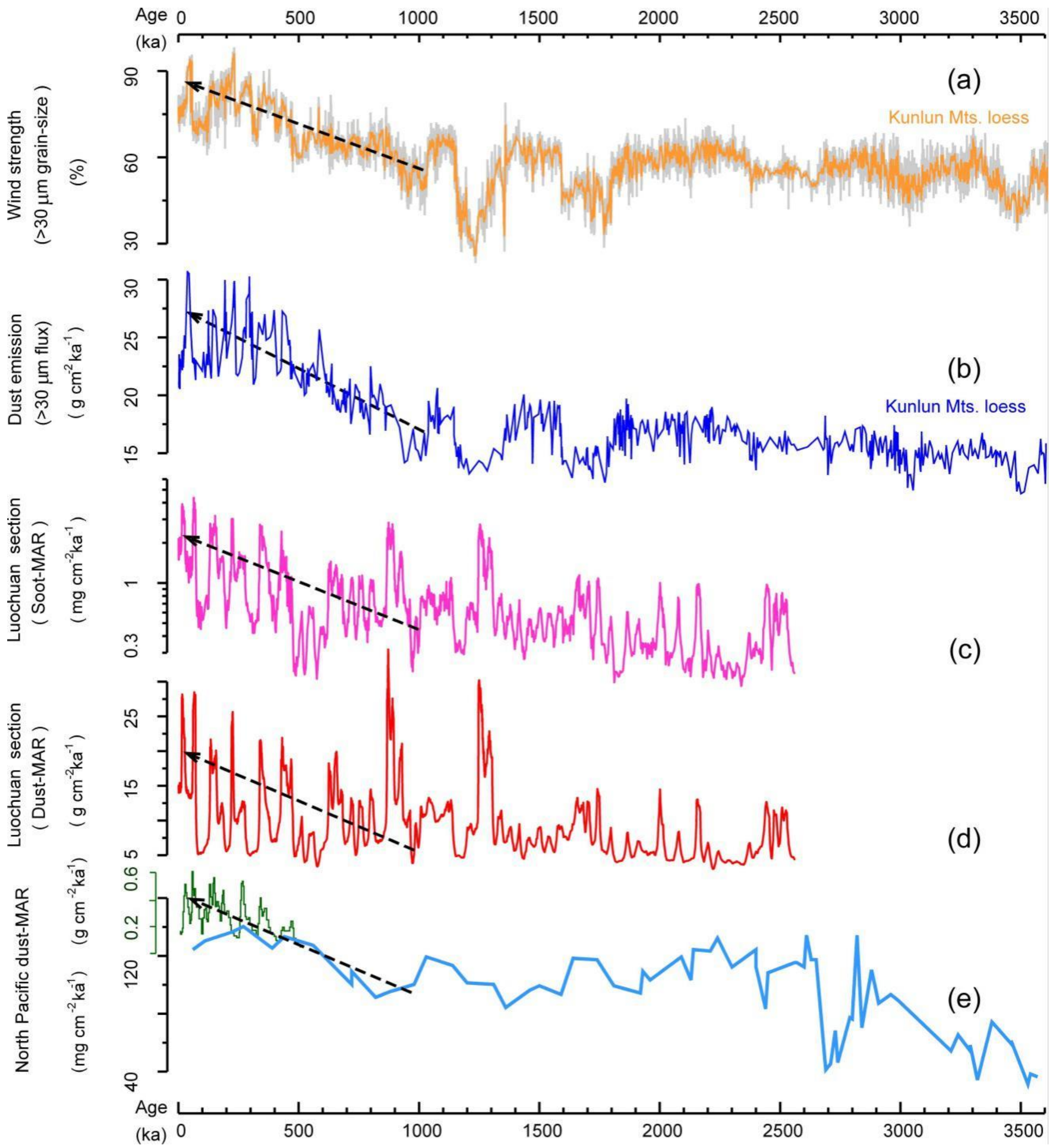

Fig. S10. Comparison of the dust grain size and flux records from the southern Tarim Basin in MLA (a, b) with soot and dust fluxes records of the Chinese Loess Plateau (6) (c, d) and dust flux records of the North Pacific Ocean (7, 8) (e).

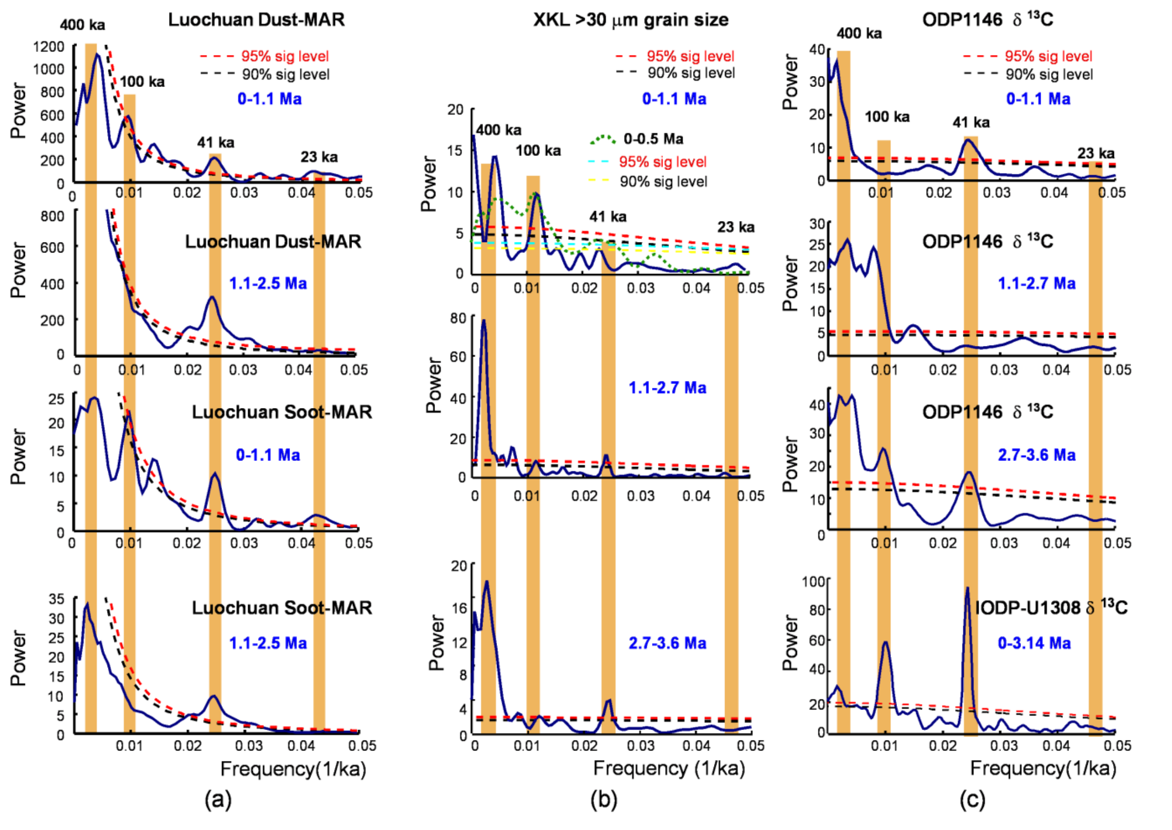

Fig. S11. Comparison of periodicities of the dust grain size record from the southern Tarim Basin in MLA (b) with dust flux and soot (big wild fire) records of the Chinese Loess Plateau (6) (a) and the carbon isotope records from the South China Sea (9) and subtropical North Atlantic Ocean (10) (c). Note all records show strong 400-ka cycles during the Plio-Quaternary.

SI References

1. Z. Shi et al., Radiative effect of mineral dust on East Asian summer monsoon during the Last Glacial Maximum: Role of snow-albedo feedback. *Geophys. Res. Lett.* **46**,https://doi.org/10.1029/2019GL084211 (2019).
2. X. M. Fang *et al*., Loess in Kunlun Mountains and its implications on desert development and Tibetan Plateau uplift in west China. *Sci. China. Ser. D Earth Sci.* **45**, 289–299 (2002).
3. J. Zachos, M. Pagani, L. Sloan, E. Thomas, K. Billups, Trends, rhythms, and aberrations in global climate 65 Ma to present. *Science* **292**, 686–693 (2001).
4. J. Laskar et al., A long-term numerical solution for the insolation quantities of the Earth. *Astron. Astrophys.* **428**, 261-285 (2004).
5. M. E. Raymo, W. F. Ruddiman, "DSDP site 607 isotope data and age models" (IGBP PAGES/World Data Center for Paleoclimatology Data Contribution Series #2004-010, National Oceanic and Atmospheric Administration, 2004).
6. Y. Han et al., Asian inland wildfires driven by glacial-interglacial climate change. *Proc. Nat. Sci. Am.* **117**, 5184-5189 (2020).
7. S. A. Hovan, D. K. Rea, N. G. Pisias, N. J. Shackleton, A direct link between the China loess and marine $\delta^{18}O$ records–Aeolian flux to the north Pacific. *Nature* **340**, 296–298 (1989).
8. D. K. Rea, H. Snoeckx, L. H. Joseph, Late Cenozoic eolian deposition in the North Pacific: Asian drying, Tibetan uplift, and cooling of the northern hemisphere. *Paleoceanography* **13**, 215-224 (1998).
9. S. C. Clemens, W. L. Prell, Y. Sun, Z. Liu, G. Chen, Southern Hemisphere forcing of Pliocene $\delta^{18}O$ and the evolution of Indo-Asian monsoons. *Paleoceanography* **23**, PA4210 (2008).

10. D. A. Hodell, J.E.T. Channell, Mode transitions in Northern Hemisphere glaciation: Co-evolution of millennial and orbital variability in Quaternary climate. *Clim. Past* **12**, 1805–1828 (2016).
